# Combining Normal/Reversed-Phase HPTLC with Univariate Calibration for the Piperine Quantification with Traditional and Ultrasound-Assisted Extracts of Various Food Spices of *Piper nigrum* L. under Green Analytical Chemistry Viewpoint

**DOI:** 10.3390/molecules26030732

**Published:** 2021-01-31

**Authors:** Mohammed H. Alqarni, Prawez Alam, Ahmed I. Foudah, Magdy M. Muharram, Faiyaz Shakeel

**Affiliations:** 1 Department of Pharmacognosy, College of Pharmacy, Prince Sattam Bin Abdulaziz University, Al-Kharj 11942, Saudi Arabia; prawez_pharma@yahoo.com (P.A.); a.foudah@psau.edu.sa (A.I.F.); 2 Department of Microbiology, College of Science, Al-Azhar University, Cairo 11884, Egypt; m.moharm@psau.edu.sa; 3 Department of Pharmaceutics, College of Pharmacy, Prince Sattam Bin Abdulaziz University, Al-Kharj 11942, Saudi Arabia; 4 Department of Pharmaceutics, College of Pharmacy, King Saud University, Riyadh 11451, Saudi Arabia; faiyazs@fastmail.fm

**Keywords:** food spices, greenness assessment, sustainable HPTLC, piperine, *Piper nigrum*

## Abstract

Due to unavailability of sustainable analytical techniques for the quantitation of piperine (PPN) in food and pharmaceutical samples, there was a need to develop a rapid and sensitive sustainable analytical technique for the quantitation of PPN. Therefore, the current research presents a fast and highly sensitive normal/reversed-phase high-performance thin-layer chromatography (HPTLC) technique with classical univariate calibration for the quantitation of PPN in various food spices of black pepper with traditional (TE) and ultrasound-assisted extracts (UBE) of various food spices of *Piper nigrum* L. under green analytical chemistry viewpoint. The amount of PPN in TE of four different spices of black pepper—namely BPMH, BPLU, BPSH, and BPPA—was found to be 309.53, 304.97, 282.82, and 232.73 mg g^−1^, respectively using a sustainable normal-phase HPTLC technique. However, the amount of PPN in UBE of BPMH, BPLU, BPSH, and BPPA was recorded as 318.52, 314.60, 292.41, and 241.82 mg g^−1^, respectively using a sustainable normal phase HPTLC technique. The greenness of normal/reversed-phase HPTLC technique was predicted using AGREE metric approach. The eco-scale was found to be 0.90, suggested excellent greenness of normal/reversed-phase technique. UBE of PPN was also found to be superior over TE of PPN. Overall, the results of this research suggested that the proposed normal/reversed-phase densitometry technique could be effectively used for the quantitation of PPN in food and pharmaceutical samples.

## 1. Introduction

The fruits of black pepper (*Piperer nigrum* L., family: Piperaceae) are used as spices and medicines [1]. The principal constituent of black pepper is piperine (PPN) (IUPAC name: (2*E*,4*E*)-5-(1,3-benzodioxol-5-yl)-1-piperidin-1-ylpenta-2,4-dien-1-one), a pungent alkaloid [2]. The other constituents like chavicine, isochavicine, and isopiperine (isomers of PPN) are also found in black pepper in very low concentrations [3,4]. The isomers of PPN are slightly pungent [3]. PPN has been studied as a potential chemotherapeutic agent in the treatment of various tumors such as melanoma, lung cancer, and colon cancer [2,5,6,7]. It also showed other therapeutic activities including antioxidant [8], anti-inflammatory [9,10], antiarthritic [10], hepatoprotective [11], antifertility [12], antiulcer [13], antifungal [14], and central nervous system depressant [15].

Various analytical techniques are available for the analysis of PPN in foods, formulations, and biological fluids. Some ultraviolet (UV) spectrometry techniques were used for the quantitation of PPN in Ayurvedic polyherbal formulations [16,17,18]. Several high-performance liquid chromatography (HPLC) techniques were also established for the quantitation of PPN in its plant extracts, foods and formulations [4,16,17,18,19,20,21,22,23,24,25]. An HPLC technique was also applied for the estimation of PPN and its isomers in egg yolk and albumin [3]. Some HPLC techniques have also been utilized for the quantitation of PPN in rat and human plasma samples [26,27]. Some liquid chromatography mass-spectrometry/mass spectrometry (LC-MS/MS) techniques were also used for the quantitation of PPN in rat and human plasma samples [28,29]. An ultra-fast liquid chromatography (UFLC) technique was also utilized for the estimation of PPN in food samples [30]. Some UFLC techniques have also been reported for the quantitation of PPN in rat and human plasma samples [31,32]. Several high-performance thin layer chromatography (HPTLC) techniques have also been applied for the estimation of PPN in its plant extract and Ayurvedic polyherbal formulations [33,34,35,36,37]. Based on the available reports on PPN analysis, the variety of analytical techniques were found for the quantitation of PPN in plant extracts, formulations, and biological samples. Nevertheless, none of those techniques are sustainable techniques. In addition, reported HPTLC techniques have also utilized toxic solvent systems for the quantitation of PPN [33,34,35,36,37]. In the recent years, the analytical techniques associated with green analytical chemistry (GAC) are increasing significantly for drug/phytochemical analysis in the literature [38,39,40,41,42].

Sustainable HPTLC techniques are completely missing in the literature for the quantitation of PPN. Different metric approaches have been reported for the greenness assessment of the analytical assays such as National Environmental Methods Index (NEMI), Analytical Eco-Scale (AES), Green Analytical Procedures Index (GAPI), Red-Green-Blue (RGB), and AGREE metric approaches [43,44,45,46,47]. The NEMI, AES, GAPI, and RGB utilizes the limited number of GAC principles [45,46,47]. However, the newly established metric approach, AGREE, utilizes all 12 principles of GAC for the greenness assessment [47]. Hence, the AGREE metric approach was utilized for the greenness assessment of present analytical techniques. The greenness profile of most of the reported analytical techniques has not been assessed evaluated properly. Based on the above assumptions, the current research was carried out to establish a fast and highly sensitive normal/reversed-phase HPTLC technique with classical univariate calibration for the quantitation of PPN in various food spices of black pepper with traditional (TE) and ultrasound-assisted extracts (UBE) of various food spices of *Piper nigrum* L. under GAC viewpoint. Four different food spices of black pepper including BPMH, BPLU, BPSH, and BPPA were studied. The normal/reversed phase HPTLC technique for the quantitation of PPN was validated for different validation parameters and evaluated for greenness profile using AGREE metric approach.

## 2. Results and Discussion

### 2.1. Method Development

Literature analysis suggested no sustainable densitometry techniques for the quantitation of PPN in plant extracts, foods, and pharmaceutical products [42]. Three isomers of PPN (chavicine, isochavicine, and isopiperine) are also present in black pepper in small amounts, but these isomers were not quantified in this study [3,4]. In this work, only PPN was quantified as it is the major compound of black pepper. In addition, these isomers did not interfere the analysis of PPN as indicated by reported HPTLC analysis [3]. No greenness profile of any reported analytical technique of PPN analysis was assessed. Accordingly, this research was conducted to establish a rapid and highly sensitive normal/reversed-phase HPTLC densitometry technique for the quantitation of PPN in various food spices of black pepper with TE and UBE of various food spices of *Piper nigrum* L. under GAC viewpoint. The utilization of sustainable densitometry techniques offers many advantages over routine analytical techniques for the quantitation of analytes in addition to reduction in the environmental toxicity [39,40].

**Figure 1:**
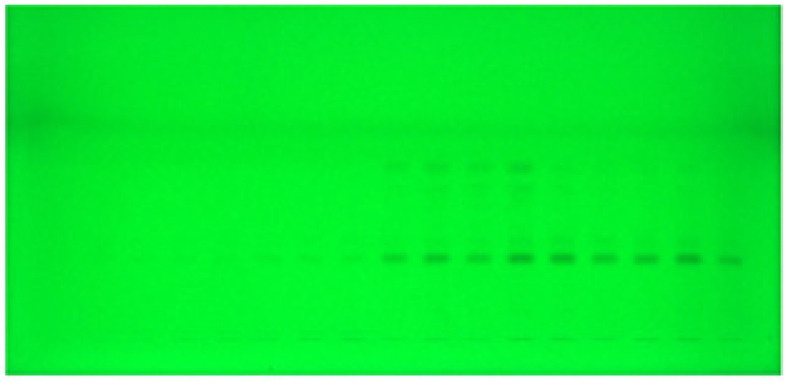
Pictorial diagram for thin-layer chromatography (TLC) plates of piperine (PPN) using sustainable normal-phase high-performance thin-layer chromatography (HPTLC).

For a normal-phase HPTLC analysis of PPN, the different proportions of cyclohexane and ethyl acetate such as cyclohexane/ethyl acetate (30:70, *v*/*v*), cyclohexane/ethyl acetate (40:60, *v*/*v*), cyclohexane/ethyl acetate (50:50, *v*/*v*), cyclohexane/ethyl acetate (60:40, *v*/*v*), and cyclohexane/ethyl acetate (70:30, *v*/*v*) were studied as the solvent systems for the development of an acceptable band for the quantitation of PPN. All the solvent systems were developed under chamber saturation conditions (Figure 1).

**Figure 2:**
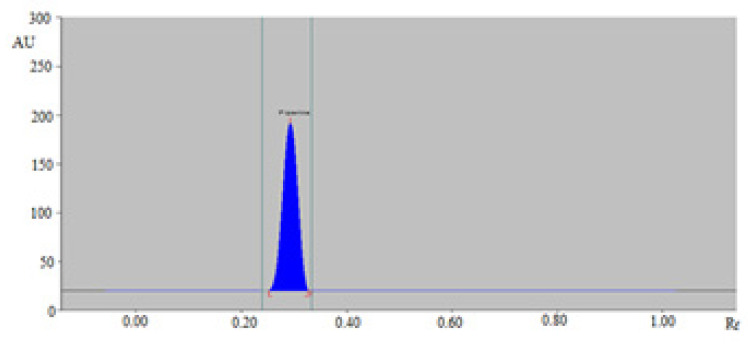
Densitometry chromatograms of standard PPN recorded using normal-phase HPTLC.

It was observed that cyclohexane/ethyl acetate (30:70, *v*/*v*), cyclohexane/ethyl acetate (40:60, *v*/*v*), cyclohexane/ethyl acetate (50:50, *v*/*v*), and cyclohexane/ethyl acetate (70:30, *v*/*v*) sustainable solvent systems offered a poor densitometry peak of PPN with poor asymmetry/tailing factor. However, cyclohexane/ethyl acetate (60:40, *v*/*v*) was found to offer a well-resolved, symmetrical, and compact densitometry peak of PPN at R_f_ = 0.29 ± 0.01 (Figure 2). Accordingly, cyclohexane/ethyl acetate (60:40, *v*/*v*) system was optimized as the sustainable solvent system for the quantitation of PPN in TE and UBE of BPMH, BPLU, BPSH, and BPPA.

For a reversed-phase HPTLC analysis of PPN, the different proportions of ethanol and water such as ethanol/water (40:60, *v*/*v*), ethanol/water (50:50, *v*/*v*), ethanol/water (60:40, *v*/*v*), ethanol/water (70:30, *v*/*v*), and ethanol/water (80:20 *v*/*v*) were studied as the solvent systems for the development of an acceptable band for the quantitation of PPN. All the solvent systems were also developed under chamber saturation conditions for a reversed-phase HPTLC technique which is shown in supplementary Figure S1. It was found that ethanol/water (40:60, *v*/*v*), ethanol/water (50:50, *v*/*v*), ethanol/water (60:40, *v*/*v*), and ethanol/water (70:30, *v*/*v*) sustainable solvent systems offered a poor densitometry peak of PPN with poor asymmetry/tailing factor. However, ethanol/water (80:20, *v*/*v*) was found to offer a well-resolved, symmetrical and compact densitometry peak of PPN at R_f_ = 0.52 ± 0.01 (Figure S2). Accordingly, ethanol/water (80:20, *v*/*v*) system was optimized as the sustainable solvent system for the quantitation of PPN in TE and UBE of BPMH, BPLU, BPSH, and BPPA.

The bands spectra for PPN for normal/reversed-phase HPTLC technique was recorded in the densitometry mode and maximum response was recorded at the wavelength (λ_max_) = 343 nm for PPN. Hence, the whole analysis of PPN was performed at λ_max_ = 343 nm using normal/reversed-phase HPTLC technique.

### 2.2. Method Validation

Various validation parameters for the quantitation of PPN were evaluated. The results for least square regression analysis for the univariate calibration curve (UCC) of PPN for a normal-phase HPTLC are included in Table 1. For a normal-phase HPTLC technique, the UCC of PPN was found to be linear in the range of 10–1000 ng band^−1^. However, the UCC of PPN was found to be linear in the range of 50–500 ng band^−1^ for a reversed-phase HPTLC technique (Table S1). The determination coefficient (R^2^) values were recorded as 0.9998 and 0.9990 for normal and reversed-phase HPTLC techniques, respectively. The R^2^ value for a normal-phase HPTLC technique was higher than a reversed-phase HPTLC technique, although both of the densitometry techniques presented good linear relationships between the concentration and peak area. In addition, the lower limit of quantification (LLOQ) and linearity range of a normal-phase HPTLC technique was much better and broader than a reversed-phase HPTLC technique. Accordingly, a normal-phase HPTLC technique was found to be more reliable for the quantitation of PPN compared to a reversed-phase HPTLC technique.

The results of the accuracy for normal-phase HPTLC technique are included in Table 2. The % recovery of PPN for a normal-phase HPTLC technique was found to be 101.40, 99.30, and 98.97% at LQC (10 ng band^−1^), MCQ (500 ng band^−1^), and HQC (1000 ng band^−1^), respectively. The %RSD in the recovery of PPN for a normal-phase HPTLC technique at LQC, MQC, and HQC was calculated as 0.98, 0.53, and 0.38%, respectively. The % recovery of PPN for a reversed-phase HPTLC technique was found to be 101.84, 100.94, and 98.82% at LQC (50 ng band^−1^), MCQ (300 ng band^−1^), and HQC (500 ng band^−1^), respectively (Table S2). The %RSD in the recovery of PPN for a reversed-phase HPTLC technique at LQC, MQC, and HQC was calculated as 1.49, 0.73, and 0.59%, respectively. The obtained values of recoveries within 100 ± 2% suggested that normal/reversed-phase HPTLC technique was accurate for the quantitation of PPN. However, a normal-phase HPTLC technique was more accurate than a reversed-phase HPTLC technique.

The results of the precision for a normal-phase HPTLC technique are included in Table 3. For a normal-phase HPTLC technique, the % RSD values of PPN for the intra-day precision were predicted as 0.78, 0.32, and 0.21% at LQC, MQC, and HQC, respectively. For a normal-phase HPTLC technique, the % RSD values of PPN for the inter-day precision were recorded as 0.86, 0.34, and 0.27% at LQC, MQC, and HQC, respectively. For a reversed-phase HPTLC technique, the % RSD values of PPN for the intra-day precision were calculated as 1.19, 1.05, and 0.80% at LQC, MQC, and HQC, respectively (Table S2). For a reversed-phase HPTLC technique, the % RSD values of PPN for the inter-day precision were calculated as 1.47, 1.13, and 0.88% at LQC, MQC, and HQC, respectively. The recorded % RSD of PPN within ±2 % suggested that normal/reversed-phase HPTLC technique was precise enough for the quantitation of PPN. However, a normal-phase HPTLC technique was more precise than a reversed-phase HPTLC technique.

The results of robustness evaluation for a normal-phase HPTLC technique are included in Table 4. The errors values in terms of % RSD for PPN after introducing small modifications in the sustainable solvent system were predicted as 0.36–0.551% for a normal-phase HPTLC technique. The R_f_ values were found to be in the range of 0.28–0.30 for a normal-phase HPTLC technique. The % RSD values of PPN were predicted as 1.14–1.15% for a reversed-phase HPTLC technique (Table S4). The R_f_ values were recorded in the range of 0.51–0.53 for a reversed-phase HPTLC technique. The minor changes in R_f_ values and lower % RSD values indicated that normal/reversed-phase HPTLC densitometry technique was robust for the quantitation of PPN. However, a normal-phase HPTLC technique was more robust than a reversed-phase HPTLC technique.

The sensitivity of PPN for normal/reversed-phase densitometry technique was calculated in terms of LOD and LOQ and results for a normal-phase HPTLC are included in Table 1. The LOD and LOQ values of PPN were calculated as 3.38 ± 0.04 and 10.14 ± 0.12 ng band^−1^, respectively for a normal-phase HPTLC technique. However, the LOD and LOQ values of PPN were recorded as 17.10 ± 0.82 and 51.30 ± 2.46 ng band^−1^, respectively for a reversed-phase HPTLC technique (Table S1). The recorded data of LOD and LOQ showed that normal/reversed-phase HPTLC technique was sensitive enough for the quantitation of PPN. However, a normal-phase HPTLC technique was highly sensitive than a reversed-phase HPTLC technique.

**Figure 3:**
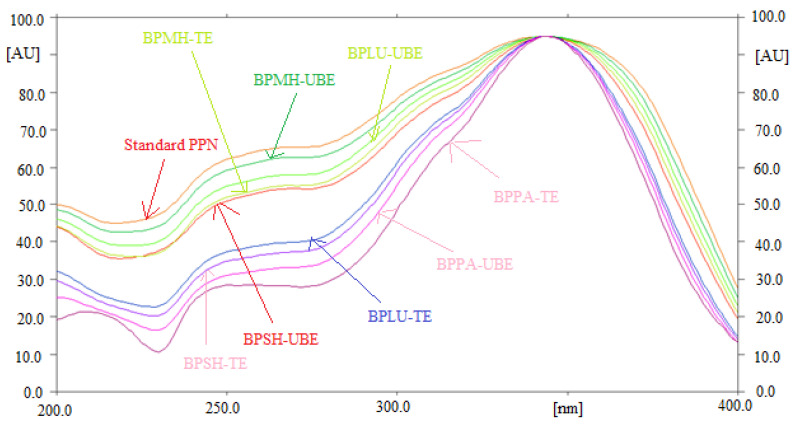
Overlaid ultra-violet (UV) absorption spectra of standard PPN and PPN in traditional and ultrasound-assisted extracts of different commercial samples of black pepper.

The specificity and the peak purity of PPN for normal/reversed-phase HPTLC technique was determined by comparing the overlaid UV absorption spectra of PPN in TE and UBE of BPMH, BPLU, BPSH, and BPPA with that of standard PPN. The overlaid UV absorption spectra of standard PPN and PPN in TE and UBE of BPMH, BPLU, BPSH, and BPPA are included in Figure 3. The maximum densitometry peak of PPN in standard and TE and UBE of BPMH, BPLU, BPSH, and BPPA were obtained at λ_max_ = 343 nm. The identical UV absorption spectra, R_f_ values, and λ_max_ of PPN in standard and TE and UBE of BPMH, BPLU, BPSH, and BPPA indicated specificity and peak purity of PPN for the quantitation of PPN using normal/reversed-phase HPTLC technique.

### 2.3. Application of Normal/Reversed-Phase HPTLC Technique for Quantitation of PPN in Different Brands of Black Pepper

**Figure 4:**
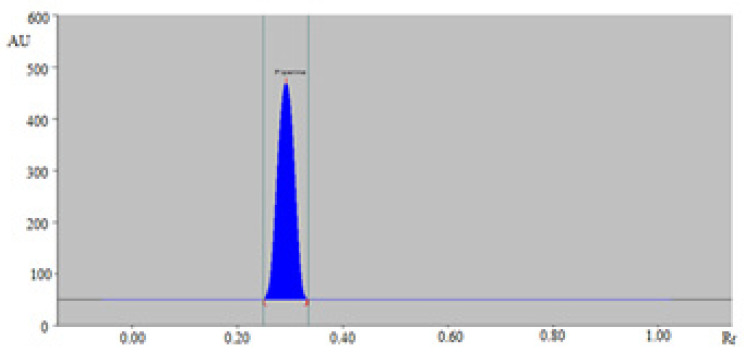
Densitometry chromatogram of PPN in methanolic extract of black pepper (BPMH) recorded using normal-phase HPTLC technique.

The sustainable normal/reversed-phase HPTLC densitometry technique could be an alternative of routine analytical techniques for the quantitation of PPN in TE and UBE of BPMH, BPLU, BPSH, and BPPA. The densitometry peaks of PPN from TE and UBE of BPMH, BPLU, BPSH, and BPPA were identified by comparing their single TLC band at R_f_ = 0.29 ± 0.01 with that of a standard PPN for normal/reversed-phase HPTLC technique. The representative normal-phase HPTLC densitogram of PPN in TE of BPMH is included in Figure 4. The densitogram in Figure 4 presented the similar peak of PPN with that of standard PPN for normal-phase HPTLC technique. The densitometry peak of PPN in TE of BPMH was also found to be similar with that of standard PPN for reversed-phase HPTLC technique (Figure S3).

**Figure 5:**
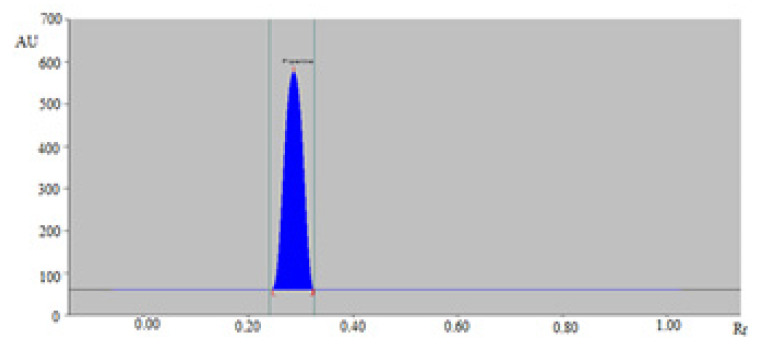
Densitometry chromatograms of PPN in methanolic extract of black pepper (BPPA) recorded using normal-phase HPTLC technique.

The representative normal-phase HPTLC densitogram of PPN in TE of BPPA is included in Figure 5. The densitograms in Figure 5 also presented the similar peak of PPN with that of standard PPN for normal-phase HPTLC technique. The densitometry peak of PPN in TE of BPPA was also found to be similar with that of standard PPN for reversed-phase HPTLC technique (Figure S4).

The content (mg g^−1^) of PPN in TE and UBE of BPMH, BPLU, BPSH, and BPPA was calculated from the UCC of PPN and results for normal-phase HPTLC are included in Table 5. The content of PPN in TE of BPMH, BPLU, BPSH, and BPPA was calculated as 309.53 ± 6.61, 304.97 ± 6.14, 282.82 ± 5.18, and 232.73 ± 4.26 mg g^−1^, respectively using a normal-phase HPTLC technique. However, the content of PPN in UBE of BPMH, BPLU, BPSH, and BPPA was recorded as 318.52 ± 6.76, 314.60 ± 6.70, 292.41 ± 5.23, and 241.82 ± 4.35 mg g^−1^, respectively using a normal-phase HPTLC technique. The content of PPN in TE of BPMH, BPLU, BPSH, and BPPA was calculated as 133.67 ± 3.02, 128.37 ± 2.95, 122.46 ± 2.64, and 119.72 ± 2.52 mg g^−1^, respectively using a reversed-phase HPTLC technique (Table S5). However, the content of PPN in UBE of BPMH, BPLU, BPSH, and BPPA was recorded as 143.84 ± 3.86, 142.29 ± 2.98, 128.39 ± 2.70, and 125.56 ± 2.57 mg g^−1^, respectively using a reversed-phase HPTLC technique (Table S5). The content of PPN was found to be highest in TE and UBE of BPMH brand using normal/reversed-phase HPTLC technique. Generally, the content of PPN was found to be significantly higher in UBE of all four brands of black pepper compared to their TE (*p* < 0.05). Based on such observations, the UBE procedure for the extraction of PPN in different brands of black pepper has been proposed as superior over its TE procedure.

The content of PPN in South African black pepper, commercial black pepper, and black pepper extract has been reported as 43, 32, and 274 mg g^−1^, respectively using an HPLC technique [17]. In another report, the content of PPN was recorded in the range of 324–497 mg g^−1^ using an HPLC technique [18]. The content of PPN analyzed using an HPLC technique was varied from 15.30–17.80 mg g^−1^ in black pepper collected from different regions of Kerala, India [21]. In another research, the content of PPN in black pepper sample was recorded in the range of 2.10–12.50 mg g^−1^ using an HPTLC technique [36]. Based on the reported data of PPN in black pepper, it was found that the content of PPN varied from country to country and regions to regions. In our research, the high contents of PPN were obtained in all four different brands of black pepper in TE and UBE using normal/reversed-phase HPTLC technique. These observations suggested that the black pepper brands available in Saudi Arabia have high contents of PPN. Overall, these results indicated that normal/reversed-phase HPTLC technique can be efficiently utilized for the quantitation of PPN in the wide variety of food and pharmaceutical products having PPN as one of the ingredients. However, a normal-phase HPTLC technique was found to be much better than a reversed-phase HPTLC technique for the quantitation of PPN in different brands of black pepper.

### 2.4. Assessment of Greenness Profiles

**Figure 6:**
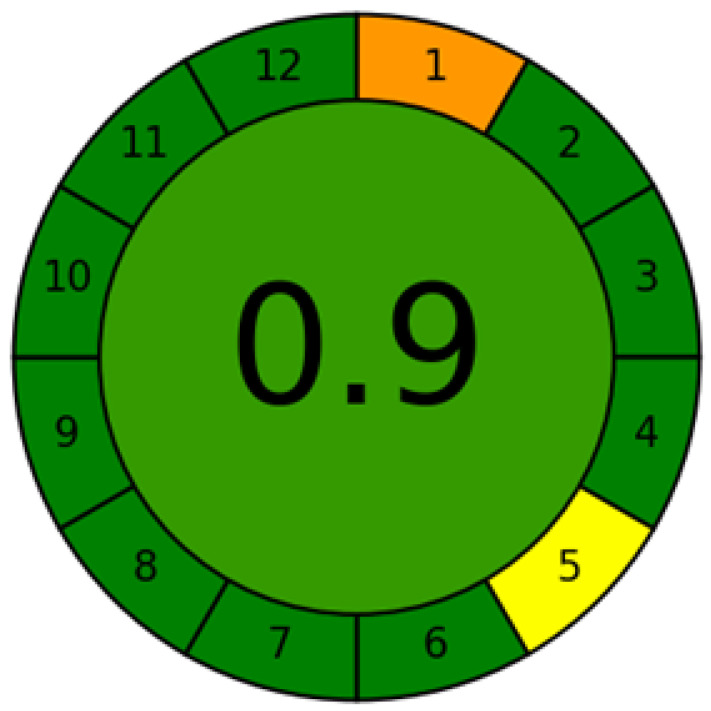
Eco-scale value of sustainable normal-phase HPTLC technique predicted using AGREE: The Analytical Greenness Calculator.

As stated previously, various metric approaches (NEMI, AES, GAPI, RGB, and AGREE) have been utilized for the assessment of greenness of the analytical procedures, but only AGREE utilized all 12 principles of GAC for the assessment of greenness [43,46,47]. Hence, in this work, the greenness of normal/reversed-phase HPTLC technique was evaluated using AGREE: The Analytical Greenness Calculator (version 0.5, Gdansk University of Technology, Gdansk, Poland, 2020). The eco-scale values for 12 different principles of GAC are assigned from 0.0–1.0 according to this metric approach [47]. The eco-scale values for a normal-phase HPTLC technique by considering 12 principles of GAC is presented in Figure 6. The eco-scale value for normal-phase HPTLC technique was predicted as 0.90. The eco-scale value for a reversed-phase HPTLC technique was also predicted as 0.90 (Figure S5). The eco-scale value of greater than 0.75 indicated the excellent greenness. The eco-scale value of 0.50 is reported as an acceptable value for greenness. An eco-scale value of less than 0.50 is associated with the unacceptability of the greenness of analytical assay [47]. Based on the eco-scale values obtained in this work, normal/reversed-phase HPTLC technique was found to have excellent greenness for the quantitation of PPN.

### 2.5. Literature Comparison

The normal/reversed-phase HPTLC technique for the quantitation of PPN was compared with various HPTLC techniques reported for the quantitation of PPN. The comparison results are included in Table 6. Three different parameters such as linearity range, accuracy, and precision of normal/reversed-phase HPTLC technique were compared with reported HPTLC techniques. The linearity range of a reported HPTLC technique was found to be 10–50 µg mL^−1^, which was much inferior to present normal-phase HPTLC (linearity range = 10–1000 ng band^−1^) and reversed-phase HPTLC (linearity range = 50–500 ng band^−1^) techniques [33]. However, the accuracy and precision of this technique were comparable to present normal/reversed-phase HPTLC technique.

The linearity range and accuracy of another HPTLC technique was also found to be inferior to present normal/reversed-phase HPTLC technique [34]. The accuracy of two reported HPTLC techniques was found to be much lower than present normal/reversed-phase HPTLC technique [35,37]. However, the linearity range of these techniques was better than present reversed-phase HPTLC technique but inferior than present normal-phase HPTLC technique [35,37]. Overall, normal/reversed-phase HPTLC technique was found to be superior than most of the reported HPTLC techniques for the quantitation of PPN. However, a normal-phase HPTLC technique was found to be highly sensitive than all reported HPTLC techniques for the quantitation of PPN.

## 3. Materials and Methods

### 3.1. Materials

Standard PPN was procured from Sigma Aldrich (St. Louis, MO, USA). Chromatography-grades methanol, ethanol, ethyl acetate, and cyclohexane were obtained from E-Merck (Darmstadt, Germany). Four different brands of black pepper coded as BPMH, BPLU, BPSH, and BPPA were purchased randomly from Hyper-market of Al-Kharj, Saudi Arabia. Chromatography-grade water was obtained from Milli-Q unit.

### 3.2. Instrumentation and Chromatographic Conditions

For the quantitation of PPN using normal/reversed-phase HPTLC technique under GAC viewpoint, the following instrumentation and chromatographic conditions were used:

- HPTLC apparatus: CAMAG TLC system (CAMAG, Muttenz, Switzerland) for normal/reversed-phase HPTLC;
- Software: WinCAT’s (version 1.4.3.6336, CAMAG, Muttenz, Switzerland) for normal/reversed-phase HPTLC;
- Syringe for sample application: CAMAG microliter Syringe (Hamilton, Bonaduz, Switzerland) for normal/reversed-phase HPTLC;
- HPTLC plate/stationary phase: 10 × 20 cm glass backed plates pre-coated with normal-phase silica gel 60 F254S plates (E-Merck, Darmstadt, Germany) for normal-phase HPTLC;
- HPTLC plate: 10 × 20 cm glass backed plates pre-coated with reversed-phase silica gel 60 F254S plates (E-Merck, Darmstadt, Germany) for reversed-phase HPTLC;
- Sample applicator: CAMAG Linomat-V (CAMAG, Muttenz, Switzerland) for normal/reversed-phase HPTLC;
- Gas for sample application: Nitrogen for normal/reversed-phase HPTLC;
- Development chamber: CAMAG automatic developing chamber 2 (ADC2) (CAMAG, Muttenz, Switzerland) for normal/reversed-phase HPTLC;
- TLC scanner: CAMAG TLC scanner-III (CAMAG, Muttenz, Switzerland) for normal/reversed-phase HPTLC;
- Mobile phase for PPN: Cyclohexane/ethyl acetate (40:60, *v*/*v*) for normal phase HPTLC;
- Mobile phase for PPN: Ethanol/water (80:20, *v*/*v*) for reversed phase HPTLC;
- Saturation time: 30 min at 22 °C for normal/reversed-phase HPTLC;
- Development distance on plate: 80 mm for normal/reversed-phase HPTLC;
- Development mode: Linear ascending mode for normal/reversed-phase HPTLC;
- Sample application rate: 150 nL/s for normal/reversed-phase HPTLC;
- Densitometry of scanning mode: Absorbance/reflectance for normal/reversed-phase HPTLC;
- Scanning wavelength for PPN: 343 nm for normal/reversed-phase HPTLC.

### 3.3. PPN UCC

The PPN stock solution (SS) was obtained separately by dissolving an accurately weighed 10 mg of PPN in 10 mL of methanol. Then, 1 mL of PPN SS was diluted further with 10 mL of methanol in order to obtain the solution of 100 μg mL^−1^. Furthermore, 1 mL of this solution was diluted again with 10 mL of methanol to get the final SS of 10 μg mL^−1^. Then, serial dilutions of PPN SS were prepared by taking variable volumes of PPN SS and diluting with methanol to obtain the concentrations in the range of 10–1000 ng band^−1^ for a normal phase HPLTLC and 50–500 ng band^−1^ for a reversed-phase HPTLC. Each solution of PPN was obtained in six replicates (*n* = 6). Approximately 200 μL of each solution of PPN was applied on HPTLC plates and the peak area was recorded for normal/reversed-phase HPTLC. The UCC for PPN was obtained by plotting the concentrations on *x*-axis against the measured area on *y*-axis, for normal/reversed-phase HPTLC [48,49]. The UCC of PPN for normal/reversed-phase HPTLC was obtained for six replicates (*n* = 6).

### 3.4. Traditional Extraction of PPN from Black Pepper Fruits

Accurately weighed 5 g of the dried fruits of different brands of black pepper (*Piper nigrum* L.) namely BPMH, BPLU, BPSH, and BPPA were refluxed with 100 mL of methanol for 1 h in a water bath and filtered via Whatman filter paper (No. 41). The marc left was refluxed again thrice with 70 mL of methanol for 1 h and filtered. The methanol was evaporated under rotary vacuum evaporator and the residue was reconstituted in 50 mL of methanol. This traditional extraction (TE) procedure was carried out in three replicates (*n* = 3). The obtained solution was used as a test solution for the quantitation of PPN in methanolic TE of different brands of black pepper using normal/reversed-phase HPTLC.

### 3.5. Ultrasound-Based Extraction of PPN from Black Pepper Fruits

The ultrasound-based extraction (UBE) of the dried fruits of BPMH, BPLU, BPSH, and BPPA was performed by ultrasound vibrations using Bransonic series (Model CPX5800H-E; Trenton, NJ, USA). An amount of 5 g of each sample of BPMH, BPLU, BPSH, and BPPA was taken and extracted using 100 mL of methanol. The methanol was evaporated under rotary vacuum evaporator and the residue was dissolved in 50 mL of methanol. The reconstituted solution was ultrasonicated at 50 °C for 1 h. The UBE was carried out in three replicates (*n* = 3). The obtained solution was used as test solution for the quantitation of PPN in UBE of different brands of black pepper using normal/reversed-phase HPTLC.

### 3.6. Method Validation

The sustainable normal/reversed-phase HPTLC-densitometry technique for the quantitation of PPN was validated in terms of linearity, precision, accuracy, robustness, sensitivity, and specificity. The linearity range of PPN was obtained by plotting the concentration of PPN against its measured HPTLC area. The linearity of PPN for a normal-phase HPTLC was evaluated in the range of 10–1000 ng band^−1^ in six replicates (*n* = 6); while, the linearity of PPN for a reversed-phase HPTLC technique was evaluated in the range of 50–500 ng band^−1^ (*n* = 6). The accuracy for PPN was predicted as the % recovery. The accuracy was obtained at lower quality control (LQC), middle quality control (MCQ), and high quality control (HQC) samples of PPN for normal/reversed-phase HPTLC technique. For a normal-phase HPTLC technique, LQC (10 ng band^−1^), MQC (500 ng band^−1^), and HQC (1000 ng band^−1^) concentrations of PPN were re-analyzed (*n* = 6). For a reversed-phase HPTLC technique, LQC (50 ng band^−1^), MQC (300 ng band^−1^), and HQC (500 ng band^−1^) concentrations of PPN were re-analyzed (*n* = 6). The % recovery for normal/reversed-phase HPTLC technique was calculated at LQC, MQC, and HQC.

The precision for PPN was evaluated as intra-day and inter-day precision. For a normal-phase HPTLC technique, intra-day precision for PPN was evaluated by the quantitation of LQC (10 ng band^−1^), MQC (500 ng band^−1^), and HQC (1000 ng band^−1^) on the same day (*n* = 6). However, inter-day precision for PPN was evaluated by the quantitation of LQC (10 ng band^−1^), MQC (500 ng band^−1^), and HQC (1000 ng band^−1^) on three different days for a normal-phase HPTLC technique (*n* = 6). For a reversed-phase HPTLC technique, intra-day precision for PPN was evaluated by the quantitation of LQC (50 ng band^−1^), MQC (300 ng band^−1^), and HQC (500 ng band^−1^) on the same day (*n* = 6). However, inter-day precision for PPN was evaluated by the quantitation of LQC (50 ng band^−1^), MQC (300 ng band^−1^), and HQC (500 ng band^−1^) on three different days for a reversed-phase HPTLC technique (*n* = 6).

The robustness for PPN quantitation was evaluated by making minor changes in the composition of mobile phase. For a normal-phase HPTLC technique, the original cyclohexane/ethyl acetate (40:60, *v*/*v*) solvent system was modified to cyclohexane/ethyl acetate (42:58, *v*/*v*) and cyclohexane/ethyl acetate (38:72, *v*/*v*) solvent systems. For a reversed-phase HPTLC technique, the original ethanol/water (80:20, *v*/*v*) solvent system was modified to ethanol/water (82:18, *v*/*v*) and ethanol/water (78:22, *v*/*v*) solvent systems. The robustness for PPN for normal/reversed-phase HPTLC technique was obtained in six replicates (*n* = 6).

The sensitivity for normal/reversed-phase HPTLC technique was evaluated in terms of detection (LOD) and quantification (LOQ) limits using a standard deviation method. The LOD and LOQ values for PPN were determined using their standard formulae reported previously in literature [39,40].

The specificity of normal/reversed-phase HPTLC technique for PPN was determined by comparing the retardation factor (R_f_) and UV-absorption spectra of PPN in commercial brands of black pepper such as BPMH, BPLU, BPSH, and BPPA with that of standard PPN.

### 3.7. Application of Normal/Reversed-Phase HPTLC Technique for Quantitation of PPN in Different Brands of Black Pepper

The prepared solutions of TE and UBE extracts of BPMH, BPLU, BPSH, and BPPA were applied on normal-phase-HPTLC plates for a normal-phase HPTLC technique and on reversed-phase-HPTLC plates for a reversed-phase HPTLC technique. The chromatograms for normal/reversed-phase HPTLC technique were recorded using the same experimental conditions as utilized for the quantitation of standard PPN. The HPTLC area of PPN in all studied solutions was obtained in three replicates (*n* = 3). The amount of PPN in all solutions was determined from the UCC of PPN (ng band^−1^) for normal/reversed-phase HPTLC technique. The ng band^−1^ concentration was converted to µg g^−1^ and finally into mg g^−1^. Hence, the final amount of PPN in four different spices of black pepper is presented in mg g^−1^.

### 3.8. Assessment of Greenness Profile

The greenness profiles of normal/reversed-phase HPTLC densitometry technique were assessed utilizing all 12 principles of GAC proposed by Pena-Pereira et al. (2020) [47]. The eco-scale values (0.0–1.0) of normal/reversed-phase HPTLC technique was obtained using AGREE: The Analytical Greenness Calculator (version 0.5, Gdansk University of Technology, Gdansk, Poland, 2020).

### 3.9. Statistical Analysis

All the values are expressed as mean ± SD of three or six replicates. The statistical analysis was carried out by applying Dunnett’s test using GraphPad Prism software (version 6, GraphPad, San Diego, CA, USA). This analysis was performed at 5% level of significance.

## 4. Conclusions

Due to the scarcity of sustainable HPTLC technique for the quantitation of PPN in literature, the present study was performed to establish a fast and highly sensitive normal/reversed-phase HPTLC technique with classical univariate calibration for the quantitation of PPN in various food spices of black pepper with TE and UBE of various food spices of *Piper nigrum* L. under GAC viewpoint. The normal-phase HPTLC technique was found to be more rapid, simple, accurate, precise, robust, and sensitive for the quantitation of PPN compared to a reversed-phase HPTLC technique. The PPN contents were found to be significantly higher in UBE of all four brands of black pepper compared to their TE. Accordingly, UBE procedure has been proposed as the preferred procedure for the extraction of PPN from black pepper. The normal-phase HPTLC technique was found to be superior and highly sensitive than reported HPTLC techniques for the quantitation of PPN. The obtained eco-scale values for normal/reversed-phase HPTLC technique indicated excellent greenness for pharmaceutical assay of PPN. After successful optimization of chromatographic runs, the normal-phase HPTLC with univariate calibration was better than another one presented at Supplementary Materials.

## Figures and Tables

**Table 1:** Results of least square regression analysis for piperine (PPN) for a sustainable normal-phase high-performance thin-layer chromatography (HPTLC) technique (mean ± SD; *n* = 6).

Parameters	Normal-Phase HPTLC
Linearity range (ng band^−1^)	10–1000
Regression equation	y = 53.29x + 579.90
R^2^	0.9998
Slope ± SD	53.29 ± 1.54
Intercept ± SD	579.90 ± 4.85
Standard error of slope	0.62
Standard error of intercept	1.98
95% confidence interval of slope	46.66–59.91
95% confidence interval of intercept	559.03–600.76
LOD ± SD (ng band^−1^)	3.38 ± 0.04
LOQ ± SD (ng band^−1^)	10.14 ± 0.12

**Table 2:** Results of accuracy evaluation for normal-phase HPTLC technique (mean ± SD; *n* = 6).

Conc. (ng Band^−1^)	Conc. Found (ng Band^−1^) ± SD	Recovery (%)	RSD (%)
10	10.14 ± 0.10	101.40	0.98
500	496.54 ± 2.67	99.30	0.53
1000	989.78 ± 3.85	98.97	0.38

**Table 3:** Results of precision evaluation for normal-phase HPTLC technique (mean ± SD; *n* = 6).

Conc. (ng Band^−1^)	Intraday Precision			Interday Precision		
	Area ± SD	Standard Error	RSD (%)	Area ± SD	Standard Error	RSD (%)
10	824 ± 6	2.44	0.78	812 ± 7	2.85	0.86
500	27,132 ± 88	35.93	0.32	26,894 ± 92	37.56	0.34
1000	53,248 ± 114	46.54	0.21	53,348 ± 148	60.43	0.27

**Table 4:** Results of robustness evaluation for normal-phase HPTLC technique (mean ± SD; *n* = 6).

Conc. (ng Band^−1^)	Mobile Phase Composition (Cyclohexane/Ethyl Acetate)			Results		
	Original	Used	Level	Area ± SD	% RSD	R_f_
		42:58	+0.2	27,894 ± 102	0.36	0.28
500	60:40	60:40	0.0	27,124 ± 126	0.46	0.29
		38:62	−0.2	26,122 ± 135	0.51	0.30

**Table 5:** Application of sustainable normal-phase HPTLC technique in determination of PPN in commercial food products in which PPN was extracted by traditional and ultrasound procedures (mean ± SD; *n* = 3).

Samples	Traditional Extraction	Ultrasound-Based Extraction
Amount of PPN (mg g^−1^)		
BPMH	309.53 ± 6.61	318.52 ± 6.76
BPLU	304.97 ± 6.14	314.60 ± 6.70
BPSH	282.82 ± 5.18	292.41 ± 5.23
BPPA	232.73 ± 4.26	241.82 ± 4.35

**Table 6:** Comparison of the current normal/reversed-phase HPTLC technique with reported techniques for the determination of PPN.

Analytical Techniques	Linearity Range	Accuracy (% Recovery)	Precision (% RSD)	Ref.
HPTLC	10–50 (µg mL^−1^)	98.13–98.18	0.02–0.12	[33]
HPTLC	18–240 (ng band^−1^)	96.50–98.57	<2.00	[34]
HPTLC	20–100 (ng band^−1^)	92.71–97.36	0.78–0.97	[35]
HPTLC	200–1000 (ng band^−1^)	97.61–98.90	0.39–0.80	[37]
Normal-phase HPTLC	10–1000 (ng band^−1^)	98.97–101.40	0.21–0.86	Present work
Reversed-phase HPTLC	50–500 (ng band^−1^)	98.82–101.84	0.80–1.47	Present work

## Data Availability

All the data associated with this article have been included in Supplementary Materials.

## Supplementary Materials

The following are available online, Figure S1: Pictorial diagram for thin-layer chromatography (TLC) plates of piperine (PPN) using sustainable reversed-phase HPTLC; Figure S2: Densitometry chromatograms of standard PPN recorded using reversed-phase HPTLC; Figure S3: Densitometry chromatogram of PPN in methanolic extract of black pepper (BPMH) recorded using reversed-phase HPTLC technique; Figure S4: Densitometry chromatograms of PPN in methanolic extract of black pepper (BPPA) recorded using reversed-phase HPTLC technique; Figure S5: Eco-scale value of sustainable reversed-phase HPTLC technique predicted using AGREE: The Analytical Greenness Calculator; Table S1: Results of least square regression analysis for piperine (PPN) for a sustainable reversed-phase high-performance thin-layer chromatography (HPTLC) technique (mean ± SD; *n* = 6); Table S2: Results of accuracy evaluation for reversed-phase HPTLC technique (mean ± SD; *n* = 6); Table S3: Results of precision evaluation for reversed-phase HPTLC technique (mean ± SD; *n* = 6); Table S4: Results of robustness evaluation for reversed-phase HPTLC technique (mean ± SD; *n* = 6); Table S5: Application of sustainable reversed-phase HPTLC technique in determination of PPN in commercial food products in which PPN was extracted by traditional and ultrasound procedures (mean ± SD; *n* = 3).
