# Supplementary material for: Combining Normal/Reversed-Phase HPTLC with Univariate Calibration for the Piperine Quantification with Traditional and Ultrasound-Assisted Extracts of Various Food Spices of Piper nigrum L. under Green Analytical Chemistry Viewpoint
Source: Molecules. 2021 Jan 31;26(3):732. doi: 10.3390/molecules26030732 (PMC7866824; doi:10.3390/molecules26030732)
Supplement: Supplementary file 1 [file molecules-26-00732-s001.pdf]

Supplementary Materials

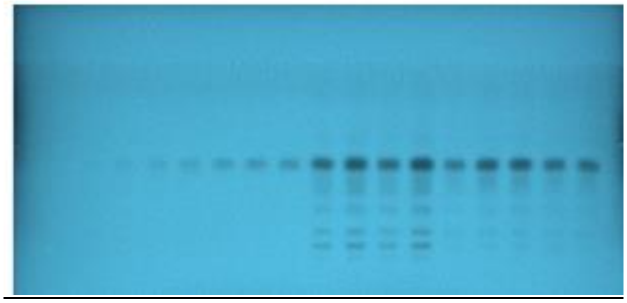

Figure S1. Pictorial diagram for thin-layer chromatography (TLC) plates of piperine (PPN) using sustainable reversed-phase HPTLC.

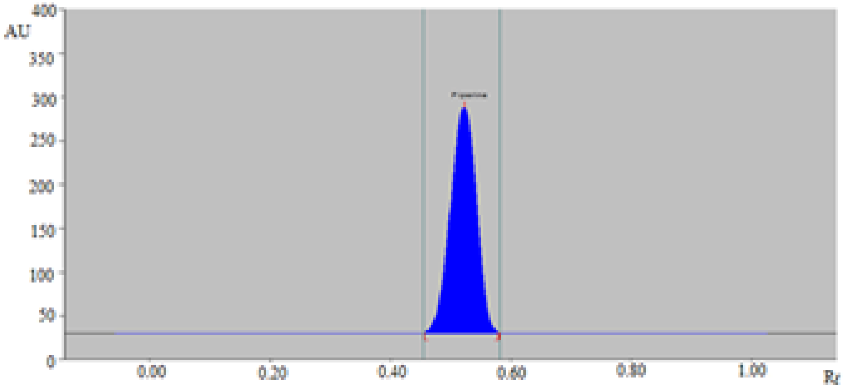

Figure S2. Densitometry chromatograms of standard PPN recorded using reversed-phase HPTLC.

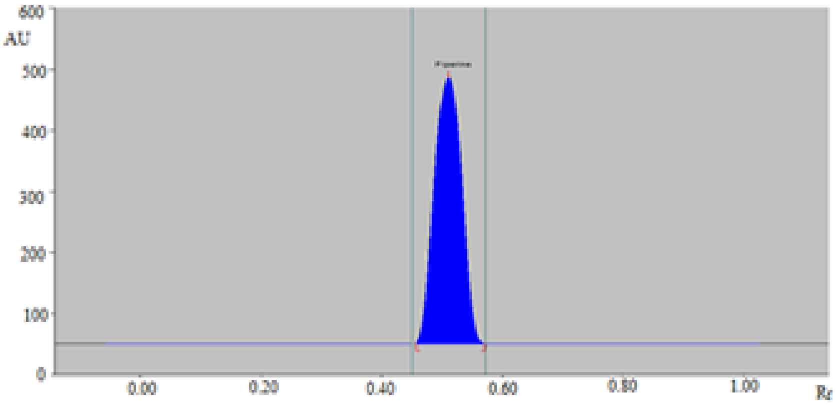

Figure S3. Densitometry chromatogram of PPN in methanolic extract of black pepper (BPMH) recorded using reversed-phase HPTLC technique.

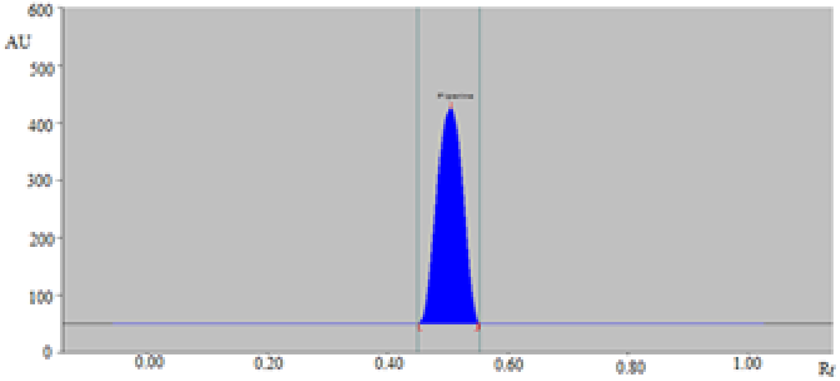

Figure S4. Densitometry chromatograms of PPN in methanolic extract of black pepper (BPPA) recorded using reversed-phase HPTLC technique.

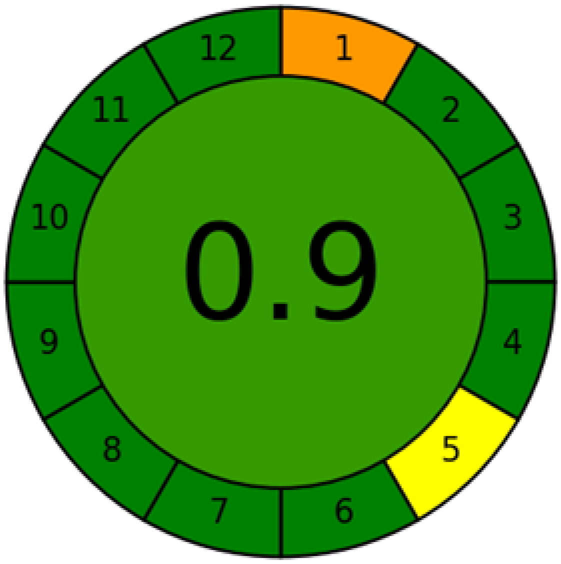

Figure S5. Eco-scale value of sustainable reversed-phase HPTLC technique predicted using AGREE: The Analytical Greenness Calculator.

Table S1. Results of least square regression analysis for piperine (PPN) for a sustainable reversed-phase high-performance thin-layer chromatography (HPTLC) technique (mean ± SD; n = 6).

Parameters	Reversed-phase HPTLC
Linearity range (ng band^{-1})	50-500
Regression equation	y = 43.27x + 522.19
R^2	0.9990
Slope ± SD	43.27 ± 1.11
Intercept ± SD	522.19 ± 4.28
Standard error of slope	0.45
Standard error of intercept	1.74
95% confidence interval of slope	38.49-48.04
95% confidence interval of intercept	503.77-540.60
LOD ± SD (ng band^{-1})	17.10 ± 0.82
LOQ ± SD (ng band^{-1})	51.30 ± 2.46

Table S2. Results of accuracy evaluation for reversed-phase HPTLC technique (mean ± SD; n = 6).

Conc. (ng band^{-1})	Conc. found (ng band^{-1}) ± SD	Recovery (%)	RSD (%)
50	50.92 ± 0.76	101.84	1.49
300	302.84 ± 2.23	100.94	0.73
500	494.14 ± 2.95	98.82	0.59

Table S3. Results of precision evaluation for reversed-phase HPTLC technique (mean ± SD; n = 6).

Conc.	Intraday precision			Interday precision		
(ng band^{-1})	Area ± SD	Standard error	RSD (%)	Area ± SD	Standard error	RSD (%)
50	2668 ± 32	13.06	1.19	2578 ± 38	15.51	1.47
300	12284 ± 129	52.67	1.05	13156 ± 149	60.84	1.13
500	20824 ± 168	68.59	0.80	21322 ± 188	76.76	0.88

Table S4. Results of robustness evaluation for reversed-phase HPTLC technique (mean ± SD; n = 6).

Conc.	Mobile phase composition (ethanol/water)			Results		
(ng band^{-1})	Original	Used		Area ± SD	% RSD	R_f
		82:18	+0.2	14134 ± 162	1.14	0.51
300	80:20	80:20	0.0	13346 ± 154	1.15	0.52
		78:22	-0.2	12356 ± 143	1.15	0.53

Table S5. Application of sustainable reversed-phase HPTLC technique in determination of PPN in commercial food products in which PPN was extracted by traditional and ultrasound procedures (mean ± SD; n = 3).

Samples	Traditional extraction	Ultrasound-based extraction
	Amount of PPN (mg g^{-1})	
BPMH	133.67 ± 3.02	143.84 ± 3.86
BPLU	128.35 ± 2.95	142.29 ± 2.98
BPSH	122.46 ± 2.64	128.39 ± 2.70
BPPA	119.72 ± 2.52	125.56 ± 2.57
